# Cartilage oligomeric matrix protein affects the biological behavior of papillary thyroid carcinoma cells by activating the PI3K/AKT/Bcl-2 pathway

**DOI:** 10.7150/jca.49144

**Published:** 2021-01-15

**Authors:** Jirong Zhang, Hongzhi Wang, Chunpeng Lv, Jun Han, Mingyu Hao, Jingjing Li, Hong Qiao

**Affiliations:** 1 Department of Geriatrics, The Second Affiliated Hospital of Harbin Medical University, Harbin 150086, Heilongjiang Province, China.; 2 Epidemiology and Health Statistics, Harbin Medical University, Harbin 150086, Heilongjiang Province, China.; 3 Department of Endocrine and Metabolism, The Fourth Affiliated Hospital of Harbin Medical University, Harbin 150086, Heilongjiang Province, China.; 4 Department of Endocrine and Metabolism, The Second Affiliated Hospital of Harbin Medical University, Harbin 150086, Heilongjiang Province, China.

**Keywords:** cartilage oligomeric matrix protein, papillary thyroid carcinoma, proliferation, invasion, apoptosis, signal pathway

## Abstract

**Objective:** To explore the effect of cartilage oligomeric matrix protein (COMP) on papillary thyroid carcinoma (PTC).

**Methods:** COMP expression levels in PTC tissues and matched adjacent normal tissues were measured using tissue microarrays. Human PTC cells were cultured and transduced with lentiviral short hairpin RNA against COMP (COMP-shRNA), a negative control (NC) shRNA, or mock transfected (Control). We used the Cell Counting Kit-8, performed colony formation assays, wound healing assays, Transwell invasion assays, flow cytometry, and measured the expression of apoptosis-related proteins at the mRNA and protein levels to explore the effects of COMP on the biological behavior of PTC cells and to discover the specific signaling pathway involved in these processes.

**Results:** COMP expression was significantly higher in PTC tissues than in adjacent normal tissues. At the cellular level, COMP promoted cell migration, increased the invasiveness of PTC cells, and inhibited apoptosis. However, differences in cell proliferation were only observed within 72 hours. At the same time, colony formation assays showed that silencing COMP inhibited the proliferation of PTC cells. We also found that COMP regulated the behavior of PTC cells by activating the PI3K/AKT/Bcl-2 pathway.

**Conclusions:** COMP is upregulated in PTC, which enhances cancer cell invasion and inhibits apoptosis, contributing to the development and progression of PTC. Thus, COMP may serve as a new biomarker for PTC.

## Introduction

Thyroid cancer is the most common malignancy of the endocrine system, accounting for over 90% of all endocrine cancer cases, and its incidence has rapidly increased worldwide over the past 30 years 1. Papillary thyroid cancer (PTC) is the most common form of thyroid cancer, accounting for approximately 90% of all thyroid cancers and 100% of all pediatric thyroid cancers 2, 3. The exact pathogenesis of PTC is not fully understood, although it is thought to be caused by radiation exposure and high iodine intake 4, 5. Some genetic mutations are highly specific for PTC, including in proto-oncogenes such as *RAS*, *BRAF*, *RET*/*PTC* and *PAX8/PPAR γ*, and have only been detected in PTC 6-8.

PTC has high rates of occurrence and recurrence 9. Early diagnosis of PTC is difficult, as many patients remain asymptomatic, and only seek medical attention due to masses or swollen lymph nodes in the neck. In a previous study, we attempted to identify new PTC-related molecular markers to facilitate early diagnosis and treatment. We have found that high cartilage oligomeric matrix protein (*COMP*) mRNA expression in PTC tissues, and that *COMP* expression was positively correlated with tumor size and lymph node metastasis 10, suggesting that COMP was involved in the occurrence of PTC.

COMP is an extracellular matrix protein and a member of the thrombospondin (TSP) family. COMP was first isolated from cartilage, and then was found to be expressed in a wide variety of tissues, including cartilage, synovium, tendons, ligaments, skin and vascular smooth muscle cells 11-15. It is mainly secreted by chondrocytes and synovial cells 16. Studies have shown that *COMP* mRNA expression may be highly correlated with tumorigenesis 17. Mounting evidence suggests that it is related to the development and progression of colon, breast, and prostate cancer 18-20. Whole COMP has been recognized as a novel biomarker for colon and breast cancer, little data exist regarding its clinical significance and biological functions in PTC.

Based on our previous experiments, we measured *COMP* expression in PTC tissues and adjacent normal tissues using tissue microarrays in this study. We explored the effects of COMP on the biological behaviors of PTC cells, i.e. proliferation, migration, invasion, and apoptosis, and uncovered the specific signaling pathway that is activated during these processes. We suggest that COMP is a new biomarker for diagnosing and treating PTC.

## Materials and methods

### Immunohistochemistry analysis

We performed immunohistochemistry to measure COMP levels in PTC tissues and adjacent normal tissues. In total, 58 pairs of PTC tissue and matched adjacent normal tissue sections were procured from Shanghai Outdo Biotech Co, Ltd. (Shanghai, China) embedded in paraffin, and incubated with anti-COMP antibody (ab11056, Abcam, Cambridge, UK; 1:1000 dilution). Samples were selected at Shanghai Outdo Biotech Co, Ltd between 2004 and 2012. Inclusion criteria: complete patient case data; no other systemic malignancy. Exclusion criteria: unqualified; non-self-matched samples; preoperative radiotherapy and chemotherapy. The study was approved by the regional ethics committee at Taizhou hospital of Zhejiang province. Two pathologists performed double-blinded slide reading with no prior knowledge of tumor grade. For every tissue section, COMP staining was observed from ten different fields of vision under a microscope, and was scored based on the percentage of positive cells and staining intensity. The percentages of stained cells among the total number of cells were scored as follows: < 5% (0 points), 5%-25% (1 point), 26%-50% (2 points), 51%-75% (3 points), and >75% (4 points). Staining intensity was scored as: not stained (0 points), light yellow (1 point), yellow-brown (2 points), and dark brown (3 points). The final staining score was obtained by multiplying these two scores, and the results were categorized as: negative (0 points), weak-positive (1-4 points), positive (5-8 points), and strong positive (9-12 points).

### Cell culture

The human PTC cell line BCPAP were purchased from Guangzhou Jennio Biological Technology Co., Ltd. (cell number: 273, Guangzhou, China), and the cell identity was validated by iCell Bioscience Inc. (Shanghai, China) using short tandem repeats. It was the fourth generation of cells. The other PTC cell line TPC-1 were provided by School of public health, Shandong First Medical University & Shandong Academy of Medical Sciences. BCPAP cells were cultured and passaged in medium containing 10% fetal bovine serum (FBS; Gibco, Grand Island, NY, USA) and TPC-1 cells were cultured and passaged in medium containing 10%FBS (BI, North America, USA), 1% penicillin/streptomycin solution (Beyotime Biotechnology Co., Ltd., Shanghai, China), and 89% RPMI 1640 (Corning Inc., Corning, NY, USA) in 37 °C incubators with 5% CO_2_. The human normal thyroid cell line HTori-3 was purchased from BeNa Culture Collection (cell number: 338687, Beijing, China) and cultured and passaged in medium containing 10% FBS (ExCell, South America) and 90% F-12K (Gibco) in 37 °C incubators with 5% CO_2_.

### Silencing COMP by lentiviral shRNA

BCPAP cells were seeded into 6-well plates at a density of 2-3 × 10^5^ cells per well for further incubation. When the cells reached 30%-50% confluence, a virus solution (Shanghai Gene Chem Co., Ltd., Shanghai, China) was added to each well to infect the BCPAP cells with a shRNA that specifically targets COMP according to the lentivirus transduction protocol with a target sequence of 5′-TGCTTGTGACAGCGATCAA-3′ and a negative control sequence of 5′-TTCTCCGAACGTGTCACGT-3′. Three types of cells were obtained: cells with silenced COMP (COMP-shRNA group), cells transfected with a non-coding shRNA (NC group), and mock transfected cells (Control group). Transfected cells were then selected with 0.75 mg/ml puromycin for 2 weeks for use in subsequent experiments.

### RNA extraction, reverse transcription (RT)-PCR, and quantitative real-time (qRT)-PCR

Total RNA was extracted from the puromycin-selected cells using TRIzol reagent, and the Roche reverse transcription kit was used to reverse transcribe RNA into cDNA. Real-time PCR was performed using the real-time PCR instrument (7500 Fast) with SYBR Green Master Mix (ROX) (Roche). Primers were provided by Sangon Biotech (Shanghai, China), and primer sequences are shown in Table 1. All experiments were performed in triplicate.

### Western blotting

Western blot analysis was performed using specific antibodies to detect the levels of COMP (ab11056, Abcam, Cambridge, UK), Bcl-2 (15071, Cell Signaling Technology, Danvers, MA, USA), Bax (2772, Cell Signaling Technology), caspase-3 (9662, Cell Signaling Technology), PI3k (4257, Cell Signaling Technology), p-PI3k p85 (ab182651, Abcam), AKT (9272, Cell Signaling Technology), p-AKT (Ser473, 4060, Cell Signaling Technology), and β-actin (Beijing Zhongshan Golden Bridge Biotechnology Co., Ltd., Beijing, China). After transfection and selection, the AKT agonist SC79 (2 µg/ml) was added to the COMP knockdown cells. Cells from each group were then collected and lysed to extract total protein after being treated in the same way for 24 h. Then 50 μg of each sample was used to determine the protein concentration by the BCA method. Sodium dodecyl sulfate-polyacrylamide gel electrophoresis was used to separate proteins, which were then transferred to polyvinylidene fluoride membranes. The membranes were subsequently incubated overnight at 4 °C with the indicated primary antibodies, and then with the appropriate secondary antibodies at room temperature for 1 h. After exposing the membranes to detection reagent, images of immunoreactive bands were taken using a Tanon gel analyzer. All western blot analyses were performed in triplicate.

### *In vitro* cell proliferation assay

The Cell Counting Kit-8 (CCK-8, Dojindo, Japan) was used to measure cell proliferation. Cells in logarithmic growth phase were collected from each group, and cell suspensions were added to a 96-well plate (100 µl per well) at approximately 9 × 10^3^, 5 × 10^3^, and 3 × 10^3^ cells per well for each group, respectively, followed by incubating the plates for 24, 48, and 72 h, respectively at 37 °C with 5% CO_2_. Freshly prepared CCK-8 solutions (10 µl) were subsequently added to the plate and incubate for 1-4 h at 37 °C. For each group, six duplicate wells were used. An ELISA analyzer was then used to measure the absorbance (OD) of each well at 450 nm, and a cell growth curve was plotted. All experiments were performed in triplicate.

### Colony formation assay

For the colony formation assays, cells in logarithmic growth phase from each group were collected, seeded into a 6-well plate at a density of 1000 cells per well, and then placed in a 5% CO_2_ incubator at 37 °C for 2 weeks. The culture medium was changed every 3 days. Cell colonies were fixed with 4% methanol, and stained with Giemsa at room temperature. The number of colonies was then counted, and all experiments were performed in triplicate.

### Wound healing assay

Cells from each group were plated into a 6-well plate at a density of 5 × 10^5^ cells per well. Once the cells reached 90%-100% confluence, a sterile pipette tip was held vertically to scratch the cell monolayer, followed by washing the cells with phosphate buffer saline. The plate was then put in the incubator after serum-free medium was added. The wound closure was monitored and imaged at 24-hour intervals using an Olympus microscope (Tokyo, Japan). Image J software was used to analyze the migration rate. This experiment was repeated at least three times for each group.

### Transwell invasion assay

The surface of the Transwell membrane (Corning Inc.) was coated with a 1:6 dilution Matrigel solution 60 µl (BD Biosciences). The Transwell was incubated with Matrigel for 30 min at 37 °C for gelling. Cell suspensions were prepared at cell densities of 5 × 10^5^ per ml. Cells were starved with culture medium containing 2% FBS for 8 h. Then 100 µl of cell suspension was added to each Transwell insert and 600 µl of 20% FBS-containing culture medium was added to the lower chamber of plate and incubated for 24 h at 37 °C. Cells on the lower surface of the membrane (migrated cells) were fixed in methanol and stained with 0.1% crystal violet. The number of migrated cells was counted from five random fields of vision at 200× magnification, and averaged to obtain the final count. All experiments were performed in triplicate.

### Flow cytometry

Cells were collected and washed with PBS. Flow cytometry was performed with the Annexin V FITC/PI apoptosis detection kit (BD Biosciences) according to the manufacturer's instructions. The samples were then analyzed with a BD FACSCalibur flow cytometer and CellQuest software (BD Biosciences, USA). Fluorescence compensation was performed using normal cells treated with apoptosis inducers as controls to correct for spectral overlap and to set the gates for cell sorting. All flow cytometry experiments were performed in triplicate.

### Statistical analysis

SPSS 19.0 software (IBM, Armonk, NY, USA) was used to analyze the data. Data that followed a normal distribution are expressed as mean ± standard deviation. A t-test was used to compare the means from two groups, and single factor variation analysis was used to compare the means of three or more groups. The F-test was performed to test the homogeneity of variance. Dunnett's T3 test was used to compare data between groups when the variance was heterogeneous, while the Least Significant Difference-t test was used for homogeneous variance comparisons. The Student-t test was used to compare data between two groups. P < 0.05 was used to indicate statistically significant differences.

## Results

### COMP was upregulated in PTC

**Figure 1:**
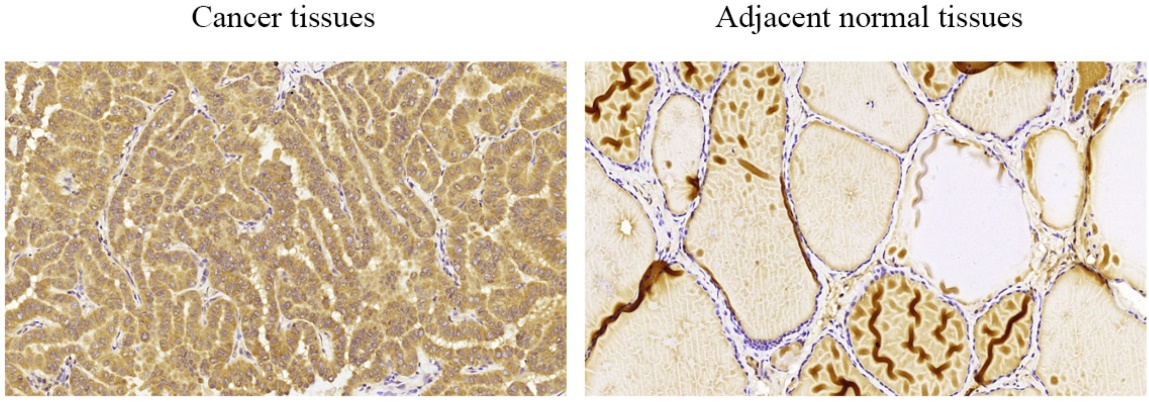
Levels of COMP expression in PTC tissues and adjacent normal tissues. Immunohistochemical analysis of COMP expression in PTC tissues and adjacent normal tissues. COMP was primarily localized in the cytoplasm of PTC cells.

We performed immunohistochemical staining of 58 pairs of PTC tissues and matched adjacent normal tissues to measure COMP expression in PTC tissues. We found that COMP was primarily localized in the cytoplasm of PTC cells (Figure 1). Among the 58 adjacent normal tissue samples, 44 (75.9%) were negative for COMP expression. In contrast, COMP expression was significantly higher in primary tumor samples, and 55 of the 58 tumor samples (94.8%) were positive for COMP staining (Table 2). These results proved that COMP is overexpressed in PTC tissues. Next, we analyzed the relationship between COMP and the clinicopathological characteristics of the 58 PTC cases (Table 3).

### COMP was overexpressed in PTC cells

**Figure 2:**
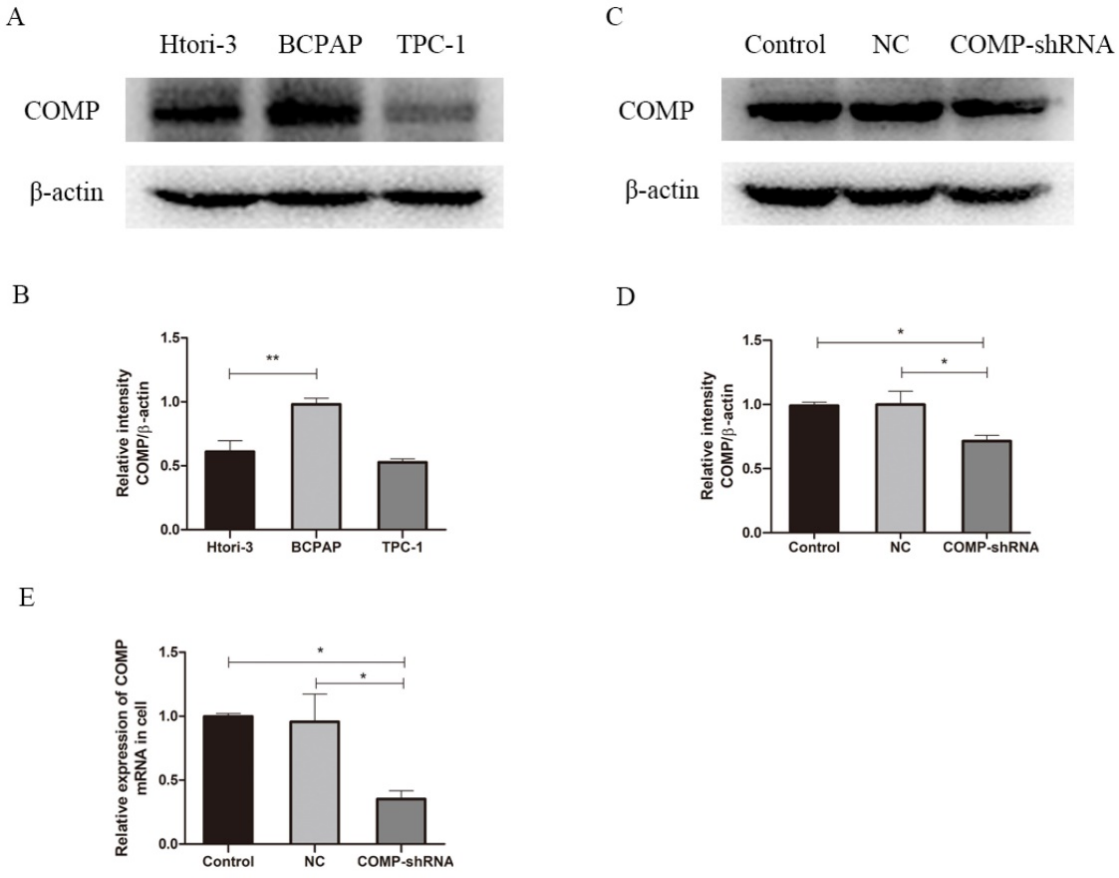
COMP expression in the human PTC cell line BCPAP. A. COMP expression was higher in BCPAP cells than in normal thyroid cells or in TPC-1 cells. COMP expression was no difference between normal thyroid cells and TPC-1 cells. B. Quantitative analysis of COMP and β-actin levels; **P < 0.01. C. COMP expression was decreased in COMP knockdown cells. D. Quantitative analysis of COMP and β-actin expression in cells transfected with mock conditions (Control), non-coding shRNA (NC), and short hairpin RNA against COMP (COMP-shRNA). E. COMP mRNA expression in PTC cells; *P < 0.05.

We performed western blot analysis to measure COMP expression in PTC cells. The PTC cell line BCPAP had significantly higher COMP expression than normal thyroid cells or TPC-1 cells (Figure 2A, *P* < 0.01). Western blot analysis showed higher COMP expression in BCPAP cells. Next, we stably transfected BCPAP cells with a lentiviral shRNA targeting COMP or empty vector to obtain COMP knockdown and control cells. qRT-PCR and western blot analysis showed that COMP expression was significantly lower in COMP-shRNA cells than in negative control and mock-transfected cells (Figure 2C and 2E).

### Effects of COMP on the proliferation of PTC cells

**Figure 3:**
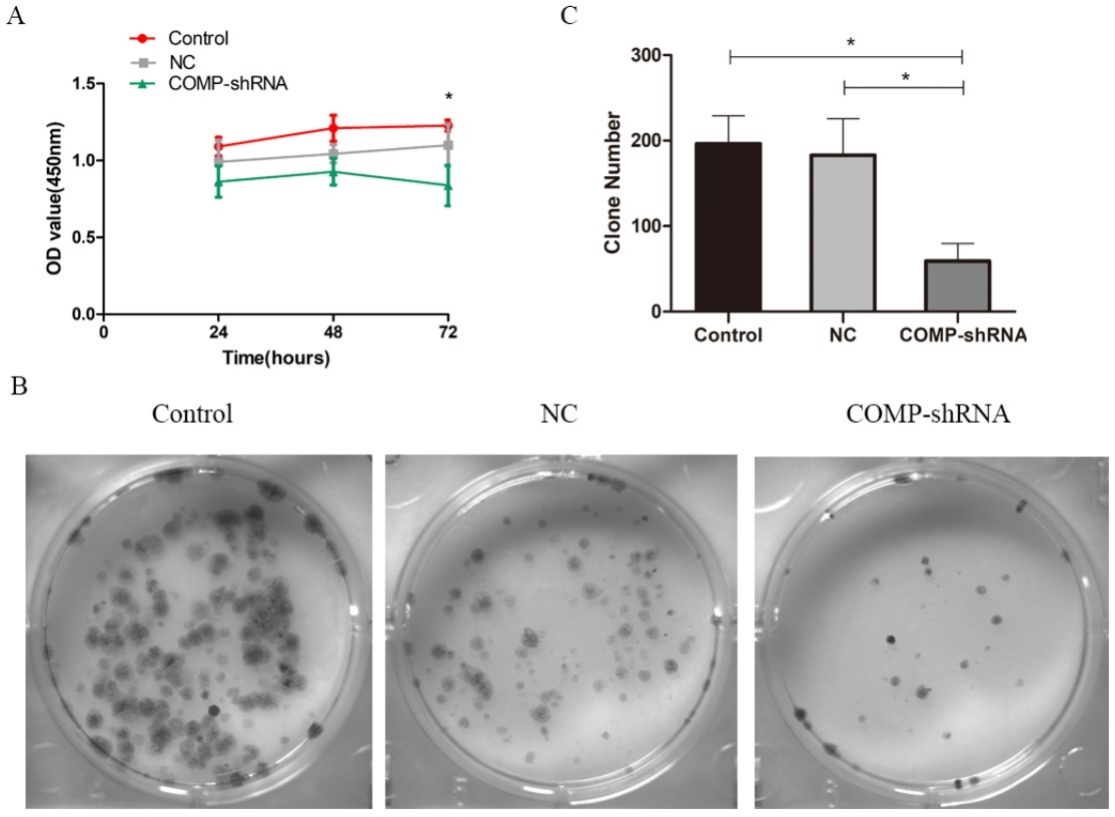
COMP promoted the proliferation of PTC cells *in vitro*. A. Cell viability was determined by CCK-8. COMP-shRNA cells had significantly lower absorbance than Control and NC cells at 72 h; *P < 0.05. B and C. Colony formation assays demonstrated that COMP promoted the growth of PTC cells. COMP-shRNA cells produced fewer colonies than Control and NC cells; *P < 0.05.

We used CCK-8 to measure the proliferation of PTC cells. Transfected cells were selected by puromycin and then monitored continuously for 72 h. COMP-shRNA cells had lower absorbance than the Control and NC cells at 24 and 48 h, but the differences were not statistically significant (all *P* > 0.05). However, at 72 h, the COMP-shRNA cells has significantly lower absorbance than the Control and NC cells (both *P*<0.05) (Figure 3A). Colony formation assays showed that the COMP-shRNA cells had significantly decreased numbers of cell colonies (Figure 3B, *P* < 0.05). These results showed that COMP promoted the proliferation of PTC cells.

### COMP enhanced the migration and invasion of PTC cells

**Figure 4:**
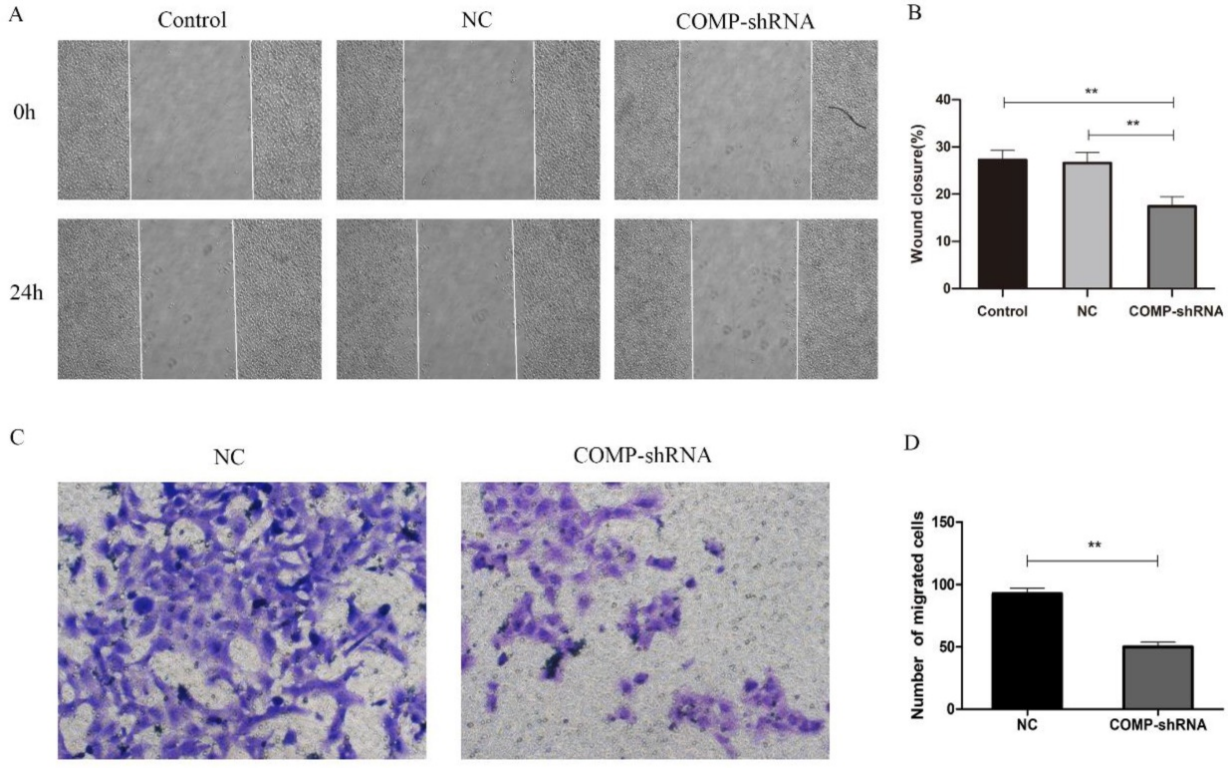
The effects of COMP on the migration and invasion of PTC cells. A. Would healing assays were performed after incubating Control, NC, and COMP-shRNA cells for 24 h. B. Wound healing assays showed significantly different cell migration rates between Control, NC, and COMP-shRNA cells; **P < 0.01. C. Transwell invasion assays using NC, and COMP-shRNA cells. D. The average number of invading cells from five random visual fields under a microscope at 200× magnification in each group; **P < 0.01.

Given the positive correlation between COMP upregulation and PTC lymph node metastasis 10, we further evaluated how COMP affected the migration and invasion of PTC cells. In terms of migration distance after 24 h as shown by wound healing assays, we found that PTC cells with COMP knockdown had significantly reduced migration compared with Control and NC cells (Figure 4A and 4B, *P* < 0.01). Transwell invasion assays showed that COMP knockdown PTC cells had significantly decreased invasiveness (Figure 4C and 4D, *P* < 0.01). An average of 50 COMP-shRNA cells showed invasion, compared with 92 in the NC groups, respectively.

### Analysis of apoptosis rates by flow cytometry

**Figure 5:**
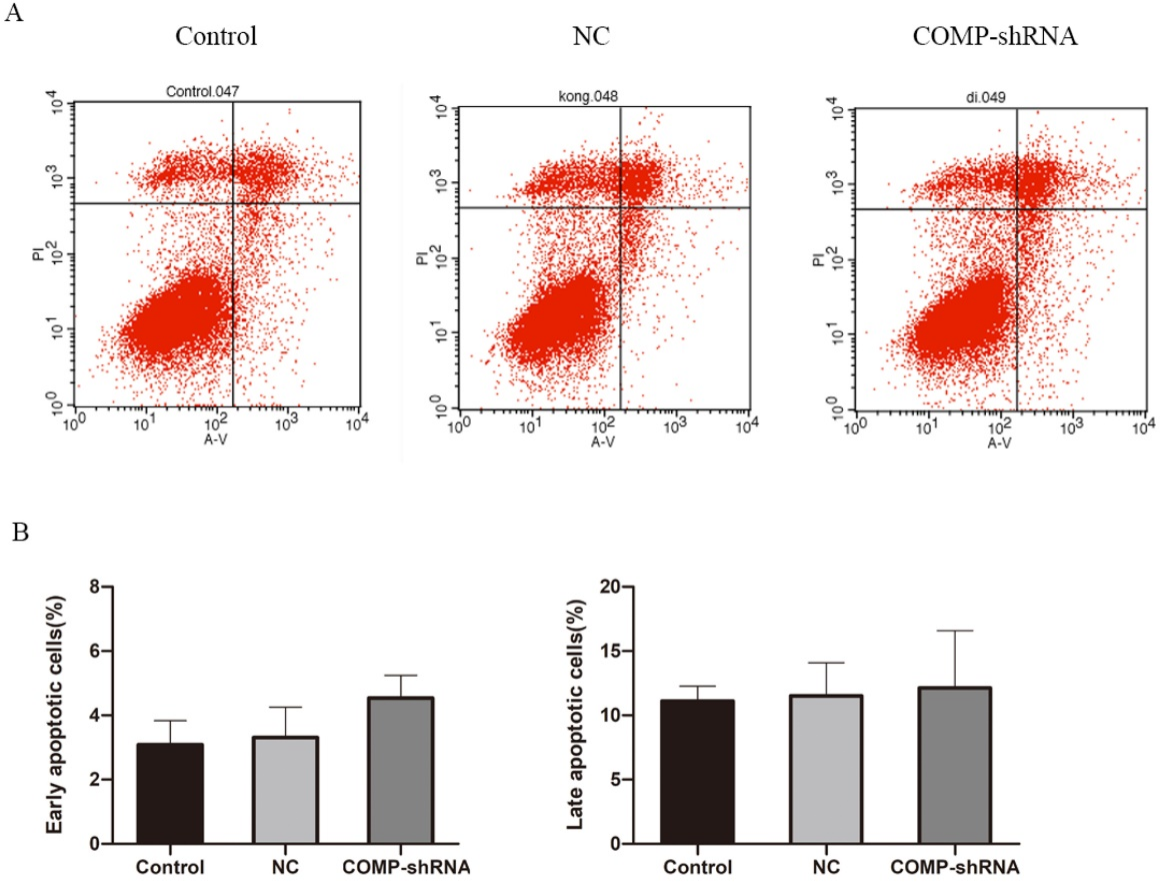
The effects of COMP on apoptosis levels by flow cytometry. A. Apoptosis levels in COMP-shRNA, Control, and NC cells. B. Apoptosis rates of COMP-shRNA, Control, and NC cells.

We next studied apoptosis rates by flow cytometry. We found that the COMP-shRNA cells had increased apoptosis rates compared with the Control and NC cells. However, early changes in apoptosis were not statistically different (Figure 5A). We plotted a bar chart based on the percentage of cells undergoing apoptosis in each group, which showed that the percentage of apoptotic COMP-shRNA cells was higher than that of control cells, but the difference was not statistically significant (Figure 5B,* P* > 0.05).

### COMP promoted PTC progression by inhibiting apoptosis

**Figure 6:**
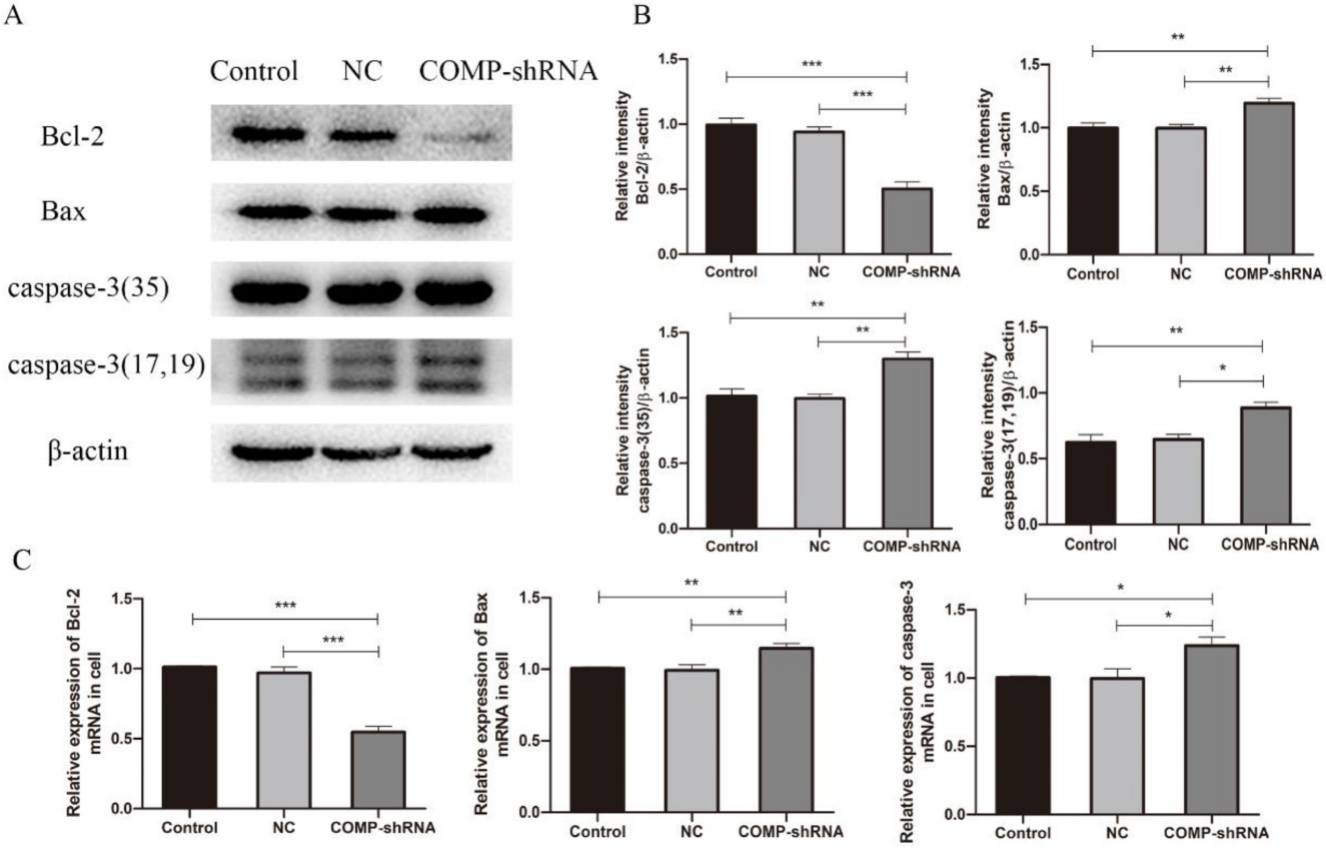
Effects of COMP on the expression of apoptosis-related proteins. A. COMP-shRNA cells had lower Bcl-2 protein levels and higher levels of Bax and caspase-3. B. Quantitative analysis of Bcl-2, Bax, caspase-3-35, caspase-3-17 and caspase-3-19 expression in Control, NC, and COMP-shRNA cells; *P < 0.05, **P < 0.01, ***P < 0.001. C. The COMP-shRNA cells had lower Bcl-2 mRNA levels and higher Bax and caspase-3 mRNA levels; *P < 0.05, **P < 0.01, ***P < 0.001.

COMP can increase the levels of apoptosis inhibitor proteins to protect chondrocytes from cell death 21. Given that apoptosis inhibition is an important feature of cancer, we measured levels of the antiapoptotic protein Bcl-2 and of the proapoptotic proteins Bax and caspase-3 to further explore the role of COMP in apoptosis of PTC cells. qRT-PCR and western blotting showed that COMP-shRNA cells had significantly lower Bcl-2 mRNA and protein levels than the Control or NC cells (Figure 6). However, COMP-shRNA cells had significantly higher Bax and caspase-3 mRNA and protein levels than Control or NC cells. There were no significant differences in the levels of these apoptosis regulators between the Control and NC cells. These results showed that COMP may play a role in PTC progression by modulating the expression of apoptosis regulators.

### COMP promoted PTC progression through the PI3K/AKT/Bcl-2 pathway

**Figure 7:**
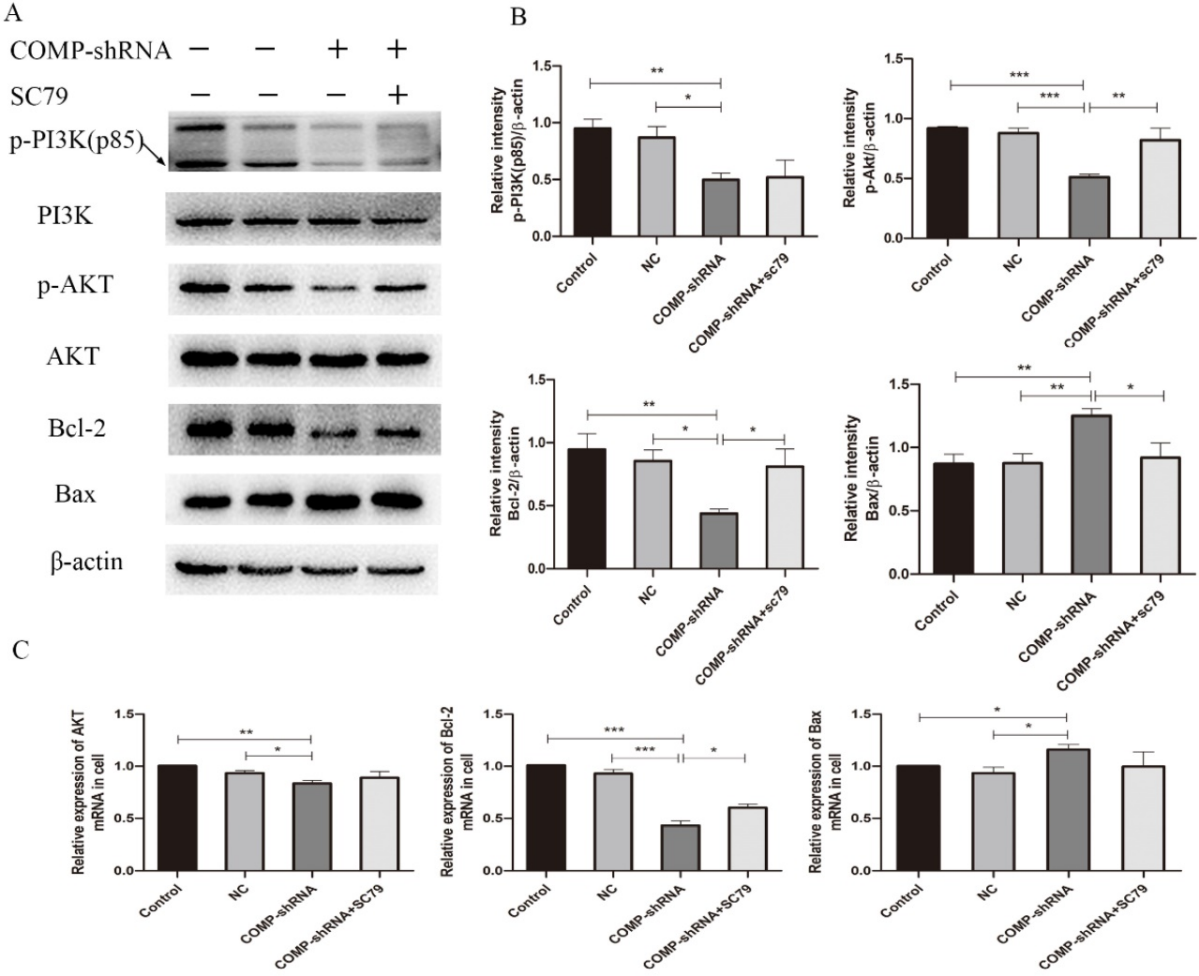
COMP activated the PI3K/AKT/Bcl-2 pathway in PTC cells. A. COMP-shRNA cells had significantly decreased levels of p-PI3K(p85), p-AKT, and Bcl-2 compared with Control and NC cells, and p-AKT and Bcl-2 levels were increased and Bax levels were decreased after SC79 was added. B. Quantitative western blot analysis of p-PI3K, p-AKT, Bcl-2 and Bax; *P < 0.05, **P < 0.01, ***P < 0.001. C. COMP-shRNA cells had significantly deceased levels of AKT and Bcl-2 mRNA; AKT and Bcl-2 expression were increased and Bax expression were decreased after SC79 was added (*P* < 0.05 for Bcl-2); *P < 0.05, **P < 0.01, ***P < 0.001.

The PI3K/AKT pathway has been shown to regulate the migration and invasion of cancer cells 22. PI3K and its downstream target AKT play important roles in promoting proliferation and inhibiting apoptosis 23. Bcl-2 is an important downstream effector of the PI3K/AKT signaling pathway and a strong negative regulator of apoptosis 24. To explore the mechanism underlying COMP inhibited cell apoptosis in PTC cells, we investigated whether COMP affected PTC progression *via* the PI3K/AKT/Bcl-2 pathway. There were no significant changes in the overall expression of PI3K and AKT. However, COMP-shRNA cells had significantly decreased levels of p-PI3K and p-AKT. After the AKT agonist SC79 was added to the COMP-shRNA cells, they showed significantly increased p-AKT and Bcl-2 levels, increased Bcl-2 mRNA expression, as well as altered levels of other apoptotic indexes (Figure 7). There were no statistically significant differences in the expression of these parameters between the Control and NC cells. These data suggested that COMP regulates the malignant behaviors of PTC cells by activating the PI3K/AKT/Bcl-2 pathway.

## Discussion

COMP is a member of the TSP family of extracellular matrix proteins that consists of five 87-kD subunits held together by interchain disulfide bonds, forming a 550-kD pentameric protein complex 25. COMP can bind to a large number of proteins, including extracellular matrix components, cell surface receptors, complement proteins, and growth factors 26. It is well known that TSPs play important roles in tumor growth, migration, and angiogenesis 27, 28. However, the roles of COMP in the development and progression of cancer as well as the mechanisms involved in these processes have not been fully clarified. Research has shown that COMP is overexpressed in colon, breast, and prostate cancers and is closely related to tumorigenesis 18-20. However, its role in PTC has not yet been established.

In this study, we further explored COMP expression in PTC tissues, and for the first time showed that COMP was primarily localized within the cytoplasm of PTC tissues. Furthermore, COMP expression was significantly higher in PTC tissues than in adjacent normal tissues. In previous study 10, we discovered four newly genes including COMP, were found to be related with PTC clinical phenotypes, and were confirmed by Spearman's correlation analyses in TCGA database. The expression level of COMP was significantly and positively related to the tumor sizes of PTC patients and the risk of lymph node metastasis. The higher the gene expression, the larger the tumor size. We analyzed the relationship between COMP and the clinicopathological characteristics of the 58 PTC cases. We found that COMP expression level was positively correlated with lymph node invasion, which was consistent with analyses in TCGA database. Therefore, we believe that COMP overexpression is significantly correlated with the occurrence of PTC and may be an independent risk factor for PTC that could be used for early diagnosis and treatment.

We detected the COMP protein expression in BCPAP cells, normal thyroid cells or TPC-1 cells. COMP is expressed in both BCPAP and TPC-1 papillary cancer-derived cell lines; however BCPAP cells, harboring the BRAFV600E mutation, have higher COMP expression level than normal thyroid cells or TPC-1 cells. These cell lines differ in their genetic background: although both are human PTC cell lines, BCPAP cells carry the V600E mutation in the BRAF gene, while TPC-1 cells harbor the RET/PTC rearrangement. BRAFV600E mutation and RET/PTC rearrangement are common genetic variations of PTC. Studies have shown that BRAFV600E mutation is the most common genetic alteration and is detected in up to 90% of PTCs 29,30. Therefore, we extended our analyses to the BCPAP cell line harboring a mutated BRAFV600E, and expressing higher level of COMP than TPC-1 cells. COMP expression in PTC could be related to the differences in their genetic background but further experiments are needed to test this hypothesis.

In this study, we explored the effects of COMP on the biological behaviors of PTC cells, including proliferation, migration, invasion, and apoptosis. We found that COMP promoted the migration and invasion of PTC cells, which is similar to its function in breast cancer cells 19; we also found that COMP was related to PTC metastasis. Consistent with the findings in breast cancer by Englund *et al*., we found that COMP had no effects on the proliferation of PTC cells *in vitro* 19. This is may be due to the short observation period of our study. However, *in vivo* experiments showed that COMP-expressing primary tumors grew faster. Similarly, Dakhova *et al.* found that prostate cancer cells with COMP knockdown showed decreased tumorigenesis 31. Our results showed that COMP knockdown only inhibited cell proliferation after 72 h. We also found that colony formation was decreased in cells with COMP knockdown, suggesting that COMP may promote tumor cell proliferation. Further animal experiments are needed to verify our findings.

Using flow cytometry, we discovered that COMP knockdown cells showed increased apoptosis. Our results were not statistically significant due to the limitation of the experimental conditions. These cells had increased levels of Bax and caspase-3, and decreased levels of Bcl-2, indicating that COMP inhibited apoptosis. Moreover, it has been proven that COMP can protect breast cancer cells from endoplasmic reticulum stress-induced apoptosis 19. Therefore, we conclude that COMP actively promotes PTC carcinogenesis by increasing migration, enhancing invasion, and inhibiting apoptosis.

In this study, we showed that COMP is a multifunctional protein with diverse functions in PTC progression. We demonstrated that COMP enhanced the invasion of PTC cells, inhibited their apoptosis, and altered their cellular metabolism, making them resistant to death, which are important characteristics of cancer cells. Previous studies have shown that COMP promotes tumor progression by altering intracellular calcium release and oxidative phosphorylation in the endoplasmic reticulum, and that COMP expression is significantly related to the time of metastasis and biochemical recurrence 20, 32. Two independent studies showed that the COMP expression in tumor cells resulted in poorer prognosis and faster spreading of primary tumors 19. Our findings suggest that COMP is related to the occurrence and metastasis of PTC, proving its harmful effects in PTC patients. COMP also has the potential to be a diagnostic biomarker for PTC, with means patients with COMP overexpression need more aggressive treatments.

To better understand the potential mechanisms that underlie the effects of COMP on PTC cells, we chose the PI3K/AKT/Bcl-2 signaling pathway because it can inhibit apoptosis. FAK binds to the SH2 domains of the PI3K p85 subunit, and this interaction plays an important role in RAS-mediated PI3K activation, which leads to further AKT phosphorylation of downstream signaling molecules. This can result in enhanced Bcl-2 transcription and suppression of apoptosis, which promotes tumor development and progression. In our efforts to investigate the molecular mechanisms through which COMP regulates the behavior of PTC cells, we found that PTC cells with COMP knockdown had decreased levels of AKT and Bcl-2 and increased levels of Bax, which is regulated by the PI3K/AKT/Bcl-2 signaling pathway. At the same time, AKT, Bcl-2, and Bax levels were further altered after SC79 was added. The mechanism underlying the regulation of apoptosis revealed by these experiments provides a theoretical basis for the use of drugs to control or inhibit apoptosis and proves that COMP could become a new target for PTC treatment.

In conclusion, COMP is upregulated in PTC, which increases cancer cell invasion and inhibits apoptosis, thus contributing to the development and progression of PTC. These results suggest that COMP could become a new biomarker and/or prognostic indicator for PTC, which could make possible early PTC diagnosis that allows for more timely treatments to improve the prognosis of PTC patients.

## Figures and Tables

**Table 1:** Fluorescence quantitative primers

Primer Name	Sequence (5'→3')
comp-f	GCAGACAAGGTGGTAGACAAGA
comp-r	GCCTGGGTCGCTGTTCATT
bcl-2-f	ACATCCATTATAAGCTGTCGCAGAGG
bcl-2-r	TGCAGCGGCGAGGTCCTG
bax-f	GATGCGTCCACCAAGAAGCTGAG
bax-r	CACGGCGGCAATCATCCTCTG
caspase-3-f	TTGAGACAGACAGTGGTGTTGATGATG
caspase-3-r	ATAATAACCAGGTGCTGTGGAGTATGC
akt-f	AGCGACGTGGCTATTGTGAAG
akt-r	GCCATCATTCTTGAGGAGGAAGT
gapdh-f	GAAGGTCGGAGTCAACGGATT
gapdh-r	CCTGGAAGATGGTGATGGGATT

**Table 2:** COMP immunohistochemical staining

Tissue sample	n	COMP expression				*P* value
		Negative (%)	Weak (%)	Positive (%)	Strong (%)	
Adjacent normal tissues	58	44 (75.9%)	14 (24.1%)			.000*
Cancer tissues	58	3 (5.2%)	19 (32.8%)	20 (34.5%)	16 (27.5%)	

*Indicates statistical difference. *P* value is based on the Chi square test.

**Table 3:** Association between clinicopathological parameters and COMP expression

Parameters	n	COMP expression				*P* value
		Negative (n=3) (%)	Weak (n=19) (%)	Positive (n=20) (%)	Strong (n=16) (%)	
**Age (years)**						0.212
<45	25	3 (12.0)	8 (32.0)	7 (28.0)	7 (28.0)	
≥45	33	0 (0.0)	11 (33.3)	13 (39.4)	9 (27.3)	
**Gender**						0.942
Male	23	1 (4.3)	8 (34.8)	7 (30.4)	7 (30.4)	
Female	35	2 (5.7)	11 (31.4)	13 (37.1)	9 (25.7)	
**Size**						0.320
≤2 cm	23	2 (8.7)	10 (43.5)	6 (26.1)	5 (21.7)	
>2 cm	35	1 (2.9)	9 (25.7)	14 (40.0)	11 (31.4)	
**Location**						0.614
Left	25	2 (8.0)	7 (28.0)	10 (40.0)	6 (24.0)	
Right	23	1 (4.3)	9 (39.1)	8 (34.8)	5 (21.7)	
Bilateral	10	0 (0.0)	3 (30.0)	2 (20.0)	5 (50.0)	
**Lymph node invasion**						0.038*
Negative	44	3 (6.8)	16 (36.4)	17 (38.6)	8 (18.2)	
Positive	14	0 (0.0)	3 (21.4)	3 (21.4)	8 (57.1)	
**T stage**						0.964
T1	32	2 (6.3)	11 (34.4)	10 (31.3)	9 (28.1)	
T2	22	1 (4.5)	6 (27.3)	9 (40.9)	6 (27.3)	
T3	2	0 (0.0)	1 (50.0)	1 (50.0)	0 (0.0)	
T4	2	0 (0.0)	1 (50.0)	0 (0.0)	1 (50.0)	
**N stage**						0.363
N0	31	2 (6.5)	7 (22.6)	12 (38.7)	10 (32.3)	
N1	27	1 (3.7)	12 (44.4)	8 (29.6)	6 (22.2)	
**AJCC stage**						0.579
I-II	38	3 (7.9)	12 (31.6)	12 (31.6)	11 (28.9)	
III-IV	20	0 (0.0)	7 (35.0)	8 (40.0)	5 (25.0)	

Pearson chi-square test. *P < 0.05 indicates a significant association among the variables.

## Abbreviations

PTC: papillary thyroid carcinoma
COMP: cartilage oligomeric matrix protein
COMP-shRNA: short hairpin RNA against COMP
TSP: thrombospondin
FBS: fetal bovine serum
qRT-PCR: quantitative real-time PCR
